# 

**Published:** 1909-09-18

**Authors:** 


### BOOKS RECEIVED.

The Panini Office, Bhuvaneshvari Ashram, India.
"The Dietetic Treatment of Diabetes." By B. D. Basu.

Hodder and Stoughton.
"Religion and Health." By Len. G. Broughton, D.D.

John Murray.
"Humours of the Country." Reprinted from *Farm and Home*.

THE

### GRADUATES' MEDICAL SCHOOLS.

DIARY FOR SEPTEMBER 20 TO 25.

### MEDICAL GRADUATES' COLLEGE AND POLYCLINIC, 22 Chenies Street, W.C.

**At 4 p.m.**

**Sept. 20**, Dr. George Pernet, **Skin**.
**Sept. 21**, Dr. C. O. Hawthorne, **Medical**.
**Sept. 22**, Mr. E. Laming Evans, **Surgical**.
**Sept. 23**, Sir Jonathan Hutchinson, **Surgical**.
**Sept. 24**, Mr. Claud Worth, **Eye**.

### ST. JOHN'S HOSPITAL FOR DISEASES OF THE SKIN, LEICESTER SQUARE, W.C.

Chesterfield Lectures.

These lectures, founded in 1895 in connection with a silver medal presented by the Earl of Chesterfield to promote the study of dermatology (and which is open for competition to those who have attended three-fourths of the lectures), are free to medical practitioners on presenting their cards and to medical students who desire to attend regularly, and will be resumed at 49 Leicester Square on Thursday evening, October 7, at 6 p.m. The Chesterfield Lecturer, Dr. Morgan Dockrell, will give his opening lecture on "The Danger to the Community of the Beauty Specialist —So-called." After each lecture demonstrations will be given on special cases, followed by clinical instruction up to eight o'clock on patients presenting themselves in the out-patient department. The lectures deal practically with diagnosis and treatment, and are illustrated by large diagrams, clinical and microscopical.

Synopsis of Lectures.—October 14 and 21, Bullous and Vesicular Eruptions. October 28, Paratuberculides (due to Tuberculous Toxins). November 4, 11, 18, and 25, Syphilis. December 2, Baldness. December 9, Fungous Diseases of the Skin. December 16, Ul-erythema.

The Metropolitan Asylums Board has made arrangements for two separate courses of lectures and demonstrations in hospital administration for the benefit of candidates for the Diploma in Public Health. They will begin respectively on the first Monday and the first Wednesday in October, and, except that they will take place at different institutions, will cover the same ground. The courses will last three months, and the lecturers are Dr. McCombie, of the North-Western Hospital, and Dr. F. M. Turner, of the South-Eastern Hospital. The fee is £3 3s.

An Appointments Board has been constituted by the University of London. The terms of reference to the Board are "to assist graduates and students of the University in obtaining appointments, and to co-ordinate and supplement the work done by the schools and institutions of the University with this object." Graduates are invited to assist in furthering the work of the Board, the aim of which is to encourage the selection of University men for all posts in the work of which the possession of a University training on scientific methods is an advantage; to assist graduates to find employment, and to assist employers to find, in the University ranks, suitable men for vacancies. It would assist the Board if, whenever graduates know of any vacant appointments suitable for University men, they would communicate with the secretary of the Board, so that he may put forward selected candidates. In this way the work of the Board as an employment exchange would be greatly helped.

A Supply of copies of the Revised Syllabus of Physical Exercises, which the Board of Education announced would be kept back until the third week in September, has now been made by the printers, and copies are procurable from the Government sale agents.

### EXHIBITION OF HYGIENE FOR THE MIDLANDS.

The Incorporated Institute of Hygiene announces that Sir William Bennett, K.C.V.O., F.R.C.S., President of the Incorporated Institute of Hygiene, will perform the ceremony of formally opening a permanent Exhibition of Hygiene on September 30, which is being established at Carlton Buildings, Paradise Street, Birmingham, under the auspices of the Midland Counties' branch of the Institute.

### CHOLERA PRECAUTIONS.

The Local Government Board have lately issued the following circular addressed to port sanitary authorities and certain riparian sanitary authorities :—

Sir,—I am directed by the Local Government Board to state that cholera, which has been, and still is, epidemic in Northern Russia, particularly in the St. Petersburg district and at Archangel, Cronstadt, Riga, and other Baltic ports, has now extended to Holland, and that several cases and deaths have already occurred at Rotterdam and in the vicinity.

The sanitary authorities of British ports trading with Rotterdam, or with North Russian ports, should be on their guard against the importation of cholera into their districts by vessels coming from places where the disease has appeared or is likely to appear. In this connection it is essential that the medical officers of health of such British ports should endeavour to keep themselves informed as to the spread of the present outbreak of cholera, and especially as to the continuance of the disease in ports where it now exists and its appearance in other ports not yet known to be affected by it. The Statement which the Board issue weekly to the medical officers of health of port and riparian sanitary authorities, and which contains information as to such cholera occurrences as have come under the Board's notice, will be of assistance in this direction.

I am to remind you that on September 9th, 1907, the Board issued a revised General Order relating to cholera, yellow fever, and plague on ships arriving from foreign ports. The Board rely on the port and riparian sanitary authorities taking all necessary steps under that Order to prevent the introduction of cholera into this country.

The Board will be glad if you will supply the medical officer of health with a copy of this Circular.

I am, Sir, your obedient Servant,
S. B. Provis, Secretary.

Local Government Board, Whitehall, S.W.
September 2, 1909.

THE HOSPITAL September 18, 1909.

*Name* ..........................................

*Address* ..........................................

..........................................

This Coupon must accompany manuscript or contributions intended for The Hospital.

